# Food Insecurity and Self-Reported Hypertension Among Hispanic, Black, and White Adults in 12 States, Behavioral Risk Factor Surveillance System, 2009

**DOI:** 10.5888/pcd11.140190

**Published:** 2014-09-18

**Authors:** Shalon M. Irving, Rashid S. Njai, Paul Z. Siegel

**Affiliations:** Author Affiliations: Rashid S. Njai, Paul Z. Siegel, Centers for Disease Control and Prevention, Atlanta, Georgia.

## Abstract

Food insecurity is positively linked to risk of hypertension; however, it is not known whether this relationship persists after adjustment for socioeconomic position (SEP). We examined the association between food insecurity and self-reported hypertension among adults aged 35 or older (N = 58,677) in 12 states that asked the food insecurity question in their 2009 Behavioral Risk Factor Surveillance System questionnaire. After adjusting for SEP, hypertension was more common among adults reporting food insecurity (adjusted prevalence ratio, 1.27; 95% confidence interval, 1.19–1.36). Our study found a positive relationship between food insecurity and hypertension after adjusting for SEP and other characteristics.

## Objective

Food security is a social determinant of health characterized by availability of and access to adequate food (1). Given the number of people with economic and physical challenges to food access, food insecurity — uncertain ability to acquire adequate food — is a public health concern (2–4). Although food insecurity is associated with hypertension among low-income populations (5), it is not known whether this relationship is independent of socioeconomic position (SEP), one potential confounder. Our objective was to examine the association between food insecurity and hypertension after adjusting for SEP and other characteristics. An independent association would support the idea that alleviating food insecurity reduces hypertension without intervening on SEP.

## Methods

We analyzed data from 12 states (Alabama, Arkansas, California, Hawaii, Illinois, Kansas, Louisiana, Nebraska, New Mexico, Oklahoma, South Carolina, and Wisconsin) that asked the food insecurity question in the 2009 Behavioral Risk Factor Surveillance System (BRFSS). BRFSS is a state-based, random-digit–dialed telephone survey of the civilian, noninstitutionalized adult US population. The sample was restricted to Hispanic, non-Hispanic black, and non-Hispanic white adults aged 35 or older (N = 58,677) because of the low prevalence of hypertension among younger adults (6) and insufficient statistical power for stratified analyses among other racial/ethnic groups.

Food insecurity was measured by asking, “How often in the past 12 months would you say you were worried or stressed about having enough money to buy nutritious meals?” Respondents answering “always,” “usually,” or “sometimes” were categorized as insecure; those answering “rarely” or “never” were categorized as secure. The food insecurity item, which measures stress-related food insecurity, was validated by the US Department of Agriculture’s Current Population Survey Food Security Supplement, a 10-item food security scale (*r* = .71, *P* < .001) (M. Nord, PhD, MS, written communication, May 2012).

Hypertension was measured by asking, “Have you ever been told by a doctor, nurse, or health professional that you have high blood pressure?” Respondents answering yes were categorized as having hypertension; those answering no or indicating borderline high blood pressure or prehypertension were categorized as nonhypertensive. Data on women reporting pregnancy-induced hypertension were excluded from analysis (6).

SEP was measured by level of education (<high school, high school graduate or GED [general education development], or any college) and a household’s relationship to the federal poverty level (ie, income as a percentage of the federal poverty level, or income-to-poverty ratio). 

Significant differences between subgroups were assessed using χ^2^ tests for categorical variables and Cochran–Mantel–Haenszel trend tests for ordinal variables. Prevalence ratios (PRs) and 95% confidence intervals (CIs) for the association between food insecurity and hypertension were calculated using logistic regression. PRs were adjusted for SEP and other potential confounders between food insecurity and hypertension: age, sex, race/ethnicity, education, percentage federal poverty level, health insurance coverage, marital status, and current smoking status (6). Analyses used SAS-callable SUDAAN (SAS version 9.3, SAS Institute Inc; SUDAAN, RTI International) to account for the BRFSS complex sampling design; predicted marginals were used to produce PR estimates (7).

## Results

Of the 58,677 adults included in the analysis, 47,734 (81.4%) were non-Hispanic white, 36,757 (62.6%) were women, and 21,248 (36.2%) were 65 or older. The prevalence of food insecurity was 17.3% (95% CI, 16.5%–18.1%) overall and varied among racial/ethnic groups — highest among Hispanics (30.3%; 95% CI, 27.1%–33.7%), lower among non-Hispanic blacks (26.7%; 95% CI, 24.5%–29.0%), and lowest among non-Hispanic whites (13.0%; 95% CI, 12.4%–13.6%) (Table 1). Prevalence of food insecurity also varied by age and sex: older adults and men were less likely to report food insecurity (both *P* < .001). The overall prevalence of self-reported hypertension was 37.4% (95% CI, 36.5%–38.3%); when stratified by race/ethnicity, the prevalence of hypertension was highest among non-Hispanic blacks (52.3%; 95% CI, 49.6%–55.0%), lower among non-Hispanic whites (36.4%; 95% CI, 35.6%–37.2%), and lowest among Hispanics (33.7%; 95% CI, 30.7%–36.8%). Prevalence of hypertension varied by age (*P* < .001); however, we found no difference by sex (*P* = .09).

**Table 1:** Prevalence of Food Insecurity and Hypertension, by Selected Sociodemographic Characteristics Among Hispanic, Non-Hispanic Black, and Non-Hispanic White Adults in 12 States^a^ (N = 58,677), Behavioral Risk Factor Surveillance System, 2009

Sociodemographic Characteristic	n^d^	Food Insecurity^b^		Self-Reported Hypertension^c^	
		Prevalence, % (95% CI)	*P* Value^e^	Prevalence, % (95% CI)	*P* Value^e^
**Overall**	58,677	17.3 (16.5–18.1)	—	37.4 (36.5–38.3)	—
**Race/ethnicity**					
Hispanic	4,067	30.3 (27.1–33.7)	<.001	33.7 (30.7–36.8)	<.001	
Non-Hispanic black	6,876	26.7 (24.5–29.0)		52.3 (49.6–55.0)		
Non-Hispanic white	47,734	13.0 (12.4–13.6)		36.4 (35.6–37.2)		
**Sex**					
Male	21,920	14.5 (13.3–15.7)	<.001	38.2 (36.8–39.6)	.09	
Female	36,757	19.8 (18.8–20.8)		36.7 (35.6–37.7)		
**Age, y**					
35–44	8,846	22.2 (20.4–24.0)	<.001	19.8 (18.2–21.5)	<.001
45–54	13,619	20.3 (18.8–21.9)		30.8 (29.1–32.5)	
55–64	14,964	15.8 (14.4–17.4)		45.1 (43.2–46.9)	
≥65	21,248	9.4 (8.6–10.3)		59.0 (57.5–60.5)	
**Marital status**					
Married/unmarried couple	34,423	14.2 (13.3–15.1)	<.001	33.6 (32.6–34.7)	<.001
Widowed	9,625	15.8 (14.2–17.5)		61.1 (58.9–63.2)	
Divorced/separated	9,832	29.8 (27.7–31.9)		41.5 (39.3–43.8)	
Never married	4,619	25.9 (22.5–29.6)		39.1 (35.6–42.6)	
**Education**					
<High school	5,792	36.0 (32.4–39.7)	<.001	44.1 (40.8–47.4)	<.001
High school graduate/GED^f^	17,855	20.3 (19.0–21.8)		41.6 (40.0–43.2)	
Any college	34,841	12.4 (11.7–13.1)		34.2 (33.2–35.2)	
**% Federal poverty level**					
<130	9,374	42.2 (39.5–44.9)	<.001	45.1 (42.5–47.6)	<.001
130 to <200	6,147	25.4 (22.9–28.0)		38.4 (35.6–41.2)	
200 to <400	20,308	11.9 (11.0–12.9)		33.9 (32.6–35.2)	
≥400	15,251	4.4 (3.9–5.1)		34.6 (33.2–36.0)	
**Health insurance coverage**					
Yes	53,001	14.5 (13.8–15.2)	<.001	37.9 (37.0–38.8)	.01
No	5,581	38.6 (35.3–42.1)		33.7 (30.5–37.0)	
**Current smoking status**					
Yes	9,247	29.8 (27.5–32.1)	<.001	37.4 (35.2–39.7)	.97
No	49,228	15.1 (14.2–15.8)		37.4 (36.4–38.3)	

Abbreviation: CI, confidence interval.

a Alabama, Arkansas, California, Hawaii, Illinois, Kansas, Louisiana, Nebraska, New Mexico, Oklahoma, South Carolina, and Wisconsin.

b Defined as a response of “always,” “usually,” or “sometimes” to the question “How often in the past 12 months would you say you were worried or stressed about having enough money to buy nutritious meals?”

c Defined as a response of yes to the question “Have you ever been told by a doctor, nurse, or other health professional that you have high blood pressure?”

d Sample sizes may vary because some respondents did not answer all questions.

e *P* values for race/ethnicity, sex, marital status, health insurance coverage, and current smoking status obtained by using χ^2 ^tests; *P* values for age, education, and percentage federal poverty level obtained by using Cochran-Mantel-Haenszel trend test.

f General educational development (GED) is a high school equivalency credential.

In the unadjusted model for the overall sample, food insecurity was associated with an increased likelihood of reporting hypertension (PR, 1.22; 95% CI, 1.15–1.30) (Table 2). This association remained after adjusting for age, sex, race/ethnicity, education, percentage federal poverty level, health insurance coverage, marital status, and current smoking status (adjusted PR, 1.27; 95% CI, 1.19–1.36).

**Table 2:** Unadjusted and Adjusted Prevalence Ratios for the Association Between Food Insecurity^a^ and Hypertension^b^, by Selected Demographic Characteristics, 12 States^c^, Behavioral Risk Factor Surveillance System, 2009

Characteristic	Unadjusted Prevalence Ratio (95% CI)	Adjusted Prevalence Ratio^d^ (95% CI)
**Overall**	1.22 (1.15–1.30)	1.27 (1.19–1.36)
**Race/ethnicity**		
Hispanic	1.39 (1.13–1.71)	1.41 (1.15–1.73)
Non-Hispanic black	1.13 (1.02–1.25)	1.13 (1.01–1.26)
Non-Hispanic white	1.17 (1.11–1.25)	1.26 (1.19–1.34)
**Sex**		
Male	1.28 (1.16–1.42)	1.35 (1.22–1.50)
Female	1.19 (1.11–1.28)	1.22 (1.13–1.31)
**Age, y**		
35–44	1.78 (1.49–2.14)	1.70 (1.41–2.05)
45–54	1.50 (1.33–1.70)	1.36 (1.19–1.55)
55–64	1.30 (1.17–1.44)	1.16 (1.02–1.32)

Abbreviation: CI, confidence interval.

a Alabama, Arkansas, California, Hawaii, Illinois, Kansas, Louisiana, Nebraska, New Mexico, Oklahoma, South Carolina, and Wisconsin.

b Defined as a response of “always,” “usually,” or “sometimes” to the question “How often in the past 12 months would you say you were worried or stressed about having enough money to buy nutritious meals?”

c Defined as a response of yes to the question “Have you ever been told by a doctor, nurse, or other health professional that you have high blood pressure?”

d Adjusted for race/ethnicity, sex, age, marital status, education, percentage federal poverty level, health insurance coverage, and current smoking status.

## Discussion

Our study shows a positive relationship between stress associated with accessing adequate food — one dimension of food insecurity — and hypertension, after adjustment for education, percentage federal poverty level, and other characteristics. Our findings suggest that food insecurity is associated with hypertension regardless of demographic group, thus, reinforcing previous research (5). Action to remove barriers to accessing affordable, healthful foods could decrease the prevalence of hypertension without directly ameliorating other factors. Thus, food security is an actionable social determinant with potential for broad public health impact (2,4,8,9). Increased access to affordable healthful foods can improve food environments and thus promote more healthful default decisions and improve healthy eating (8,10). Healthier lifestyles create the potential for reduced risk of hypertension and other chronic diseases (3).

The BRFSS has some limitations. BRFSS data are self-reported and cross-sectional and thus are subject to issues inherent in self-reported data and do not allow explorations of causality. Second, the BRFSS assesses lifetime prevalence of hypertension and 12-month prevalence of food insecurity. Thus, if food security occurred more than 12 months before the survey, survey data may underestimate the association between hypertension and food insecurity. Finally, the generalizability of the findings is limited because only 12 states used the food security question in their BRFSS. However, our study sample (adults aged 35 or older who were asked the food insecurity question) represents 30% of the US population aged 35 or older (11). Despite these limitations, the validated single-item BRFSS food security question can be used to improve efforts to monitor prevalence and assess the effectiveness of interventions.

Addressing the relationship between food insecurity and hypertension or other cardiovascular diseases enhances our understanding of effects associated with the stress of attempting to meet one’s basic needs. Whether food insecurity is caused by limited socioeconomic access, physiological stress, or overconsumption of nutrient-poor, high-sodium food (4), our findings underscore the importance of examining food insecurity further in the context of chronic disease prevention.

## References

[1] Hamm MW , Bellows AC . Community food security: background and future directions. J Nutr Educ Behav 2003;35(1):37–43. 10.1016/S1499-4046(06)60325-4 12588679

[2] Coleman-Jensen A , Nord M , Singh A. Household food security in the United States, 2012. US Department of Agriculture, Economic Research Service; 2013.

[3] National Prevention Council. National prevention council action plan: implementing the national prevention strategy. Washington (DC): US Department of Health and Human Services, Office of the Surgeon General, 2012.

[4] Seligman HK , Schillinger D . Hunger and socioeconomic disparities in chronic disease. N Engl J Med 2010;363(1):6–9. 10.1056/NEJMp1000072 20592297

[5] Seligman HK , Laraia BA , Kushel MB . Food insecurity is associated with chronic disease among low-income NHANES participants. J Nutr 2010;140(2):304–10. 10.3945/jn.109.112573 20032485 PMC2806885

[6] Gillespie CD , Hurvitz KA . Prevalence of hypertension and controlled hypertension — United States, 2007–2010. MMWR Surveill Summ 2013;62(3):144–8. 24264505

[7] Bieler GS , Brown GG , Williams RL , Brogan DJ . Estimating model-adjusted risks, risk differences, and risk ratios from complex survey data. Am J Epidemiol 2010;171(5):618–23. 10.1093/aje/kwp440 20133516

[8] Frieden TR . A framework for public health action: the health impact pyramid. Am J Public Health 2010;100(4):590–5. 10.2105/AJPH.2009.185652 20167880 PMC2836340

[9] Chilton M , Rose D . A rights-based approach to food insecurity in the United States. Am J Public Health 2009;99(7):1203–11. 10.2105/AJPH.2007.130229 19443834 PMC2696644

[10] Young CR , Aquilante JL , Solomon S , Colby L , Kawinzi MA , Uy N , Improving fruit and vegetable consumption among low-income customers at farmers markets: Philly Food Bucks, Philadelphia, Pennsylvania, 2011. Prev Chronic Dis 2013;10:E166. 10.5888/pcd10.120356 24135390 PMC3804016

[11] US Census Bureau. Census summary: 2000: Census 2000 profile. www.census.gov/prod/2002pubs/c2kprof00-us.pdf. Accessed February 3, 2014.

